# Evidence of Interaction between Polychlorinated Biphenyls and Phthalates in Relation to Human Sperm Motility

**DOI:** 10.1289/ehp.7305

**Published:** 2005-01-10

**Authors:** Russ Hauser, Paige Williams, Larisa Altshul, Antonia M. Calafat

**Affiliations:** ^1^Department of Environmental Health, Occupational Health Program, Harvard School of Public Health, Boston, Massachusetts, USA; ^2^Vincent Memorial Obstetrics and Gynecology Service, Andrology Laboratory and in Vitro Fertilization Unit, Massachusetts General Hospital, Boston, Massachusetts, USA; ^3^Department of Biostatistics, Harvard School of Public Health, Boston, Massachusetts, USA; ^4^Department of Environmental Health, Environmental Science and Engineering Program, Harvard School of Public Health, Boston, Massachusetts, USA; ^5^National Center for Environmental Health, Centers for Disease Control and Prevention, Atlanta, Georgia, USA

**Keywords:** environmental, epidemiology, human, sperm, synergy

## Abstract

Previously, we reported evidence of inverse associations between exposure to some polychlorinated biphenyls (PCBs) and some phthalate monoesters in relation to semen parameters, specifically sperm motility. Because humans are exposed to both phthalates and PCBs and because experimental studies suggest that PCBs may interact with glucuronidative enzymes that are responsible for phthalate metabolism, we explored the potential interaction between phthalates and PCBs in relation to human semen quality. We studied 303 men who were partners in subfertile couples seeking infertility diagnosis from the andrology laboratory at Massachusetts General Hospital. Semen parameters were dichotomized based on World Health Organization reference values, and phthalate and PCB levels were dichotomized at their respective medians. After adjusting for age and abstinence time, for below reference sperm motility there was a greater than additive interaction between monobenzyl phthalate and PCB-153 [relative excess risk due to interaction (RERI) = 1.40; 95% confidence interval (CI), 0.41–3.22], sum of PCBs (RERI = 1.24; 95% CI, 0.15–2.94), and cytochrome P450 (CYP450)-inducing PCBs (RERI = 1.30; 95% CI, 0.21–3.06). For below-reference sperm motility, there was also a greater than additive interaction between monobutyl phthalate (MBP) and PCB-153 (RERI = 1.42; 95% CI, 0.09–3.76) and CYP450-inducing PCBs (RERI = 1.87; 95% CI, 0.56–4.52) and a suggestive interaction between MBP and sum of PCBs (RERI = 1.35; 95% CI, −0.11 to 3.48). In conclusion, because there are important risk assessment and public health implications of interactions between these two ubiquitous classes of compounds, further studies need to be conducted to confirm these results and identify potential mechanisms of interactions.

Environment–environment, gene–environment, and gene–gene interactions play important roles in determining the relationship between exposure to environmental chemicals and risk to human health. Traditionally, human studies have focused on health effects associated with exposure to a single environmental chemical. These studies have been useful for identifying human health risks from exposure to environmental chemicals. However, exploration of interactions among environmental chemicals is an important area of inquiry because it is well appreciated that humans are exposed to a large number of chemicals both concurrently and sequentially, from multiple sources and through various routes of exposure (Suk et al. 2002).

Currently, there is scientific and public concern about potential human health risks from exposure to phthalates, diesters of phthalic acid. These concerns stem from studies showing that most of the U.S. general population is exposed to phthalates (Silva et al. 2004), as well as from animal studies suggesting that some phthalates are developmental and reproductive toxicants (Agarwal et al. 1985; Cater et al. 1977; Foster et al. 1980; Mylchreest et al. 2000; Park et al. 2002). A recent report on levels of phthalate monoester metabolites in urine samples collected for the National Health and Nutrition Examination Survey (NHANES) 1999–2000 (Silva et al. 2004) showed that four phthalate metabolites, namely, monoethyl phthalate (MEP), mono-2-ethylhexyl phthalate (MEHP), monobutyl phthalate (MBP), and monobenzyl phthalate (MBzP), were present in more than 75% of subjects sampled. Monoester phthalate metabolites were measured because of potential sample contamination from the precursor diesters and because the metabolites are considered the biologically active toxicant (Li et al. 1998; Peck and Albro 1982).

Polychlorinated biphenyls (PCBs) are a family of synthetic, persistent, lipophilic, poly-aromatic chemicals found in the general U.S. population [Centers for Disease Control and Prevention (CDC) 2003]. Laboratory animal and limited human studies suggest possible associations between PCBs and semen abnormalities (Bush et al. 1986; Dallinga et al. 2002; Hauser et al. 2003; Richthoff et al. 2003). The half-life of PCBs in blood ranges from 1 to ≥10 years (Phillips et al. 1989), depending on congener type. Therefore, serum levels of PCBs, which are an integrated measure of internal dose, reflect exposure from all sources over the previous years. Although the use and manufacture of PCBs in the United States were banned nearly 30 years ago, PCBs are ubiquitous and persist in the environment. The general population continues to be exposed to PCBs primarily through ingestion of contaminated foods (fish, meat, and dairy products).

In previous publications, we reported suggestive evidence of inverse associations between exposure to select PCB congeners [i.e., PCB-138, sum of PCBs (∑PCBs), and PCBs classified as enzyme inducers] and semen parameters (i.e., sperm motility and morphology) (Hauser et al. 2003), as well as inverse associations between exposure to select phthalate metabolites (i.e., MBP and MBzP) and sperm concentration and motility (Duty et al. 2003).

Silva et al. (2003a) recently found inter-individual differences in the percentage of free phthalate monoesters in urine, a likely reflection of interindividual differences in phthalate glucuronidation. These authors hypothesized that individual differences in glucuronidative capacity, as measured by the urinary levels of free phthalate monoesters, may determine a person’s susceptibility to phthalate toxicity. Differences in glucuronidative capacity may be a result of genetic polymorphisms in uridine 5′-diphosphate glucuronosyltransferase (UDP-GT) or potential inhibition of UDP-GT, the enzyme responsible for glucuronidation of phthalate monoesters. A recently published study showed that hydroxylated PCBs (OH-PCBs) inhibited the glucuronidation of 3-hydroxy benzo[*a*]pyrene by inhibiting UDP-GT (van den Hurk et al. 2002). On the basis of the results of Silva et al. (2003a, 2004) and van den Hurk et al. (2002), we conducted an exploratory analysis of the potential statistical interaction between serum and urinary levels of PCBs and phthalates, respectively, in an ongoing study on human semen quality.

## Materials and Methods

This study was approved by the Harvard School of Public Health and Massachusetts General Hospital human subjects committees. All subjects signed an informed consent after receiving an explanation about the study procedures and their possible consequences. Study subjects were men who were partners in subfertile couples seeking infertility diagnosis from the Vincent Burnham Andrology Laboratory at Massachusetts General Hospital between January 2000 and April 2003. One spot urine sample and one blood sample were collected from each subject on the same day as the semen sample.

Additional details of subject recruitment and the semen analysis methodologies have been described previously (Hauser et al. 2003). Briefly, recruitment of eligible participants in the present study has been ongoing since January 2000. As in our earlier studies (Duty et al. 2003; Hauser et al. 2003), sperm counts and motility were measured by computer-aided semen analysis using the Hamilton Thorne IVOS 10 Analyzer (Hamilton-Thorne Research, Beverly, MA). Sperm morphology was scored using the strict criteria (Kruger et al. 1988).

The analytical approach for the analysis of urine for levels of phthalate metabolites has been described in detail elsewhere (Silva et al. 2003b). Briefly, phthalate metabolite determination in urine involved enzymatic deconjugation of the metabolites from the glucuronidated form and then solid-phase extraction followed by detection using reversed-phase high-performance liquid chromatography–isotope dilution tandem mass spectrometry. Detection limits were in the low nanogram per milliliter range. One method blank, two quality control samples (human urine spiked with phthalates), and two sets of standards were analyzed along with every 21 unknown urine samples. Analysts at the CDC were blind to all information concerning subjects. Urinary phthalate levels (in nanograms per milliliter) were normalized for dilution by specific gravity adjustment. Specific gravity was measured using a handheld refractometer (National Instrument Company, Inc., Baltimore, MD), which was calibrated with deionized water before each measurement.

Blood samples were analyzed for PCBs by the Organic Chemistry Analytical Laboratory at the Harvard School of Public Health. The analytical approach has been described in detail elsewhere (Korrick et al. 2000). Briefly, the serum extracts were analyzed by gas chromatography (GC) with electron capture detection (ECD) using a Hewlett-Packard 5890 Series II GC with a fused silica capillary column (DB5, 30 m × 0.25 mm, 0.25 μm; J&W Scientific, Folsom, CA). Confirmatory analysis was performed using a Hewlett-Packard 6980 GC with a micro-ECD and capillary column of different polarity. Quantitation was based on the response factors of individual PCB congeners relative to the internal standard (PCB-166 by International Union of Pure and Applied Chemistry nomenclature). PCB concentrations (in nanograms per gram lipid) were reported as individual congeners and as the sum of all congeners measured (∑PCBs). The amount of each PCB congener in samples was corrected by the amount of that analyte in the procedural blank associated with the analytical batch. Results were not adjusted for surrogate recoveries. The percent lipid for each serum sample was measured gravimetrically, by weighing an aliquot of sample extract evaporated to dryness.

### Statistical analysis.

In our previous studies (Duty et al. 2003; Hauser et al. 2003), the strongest relationships were found between sperm motility and MBP, MBzP, ∑PCBs, and PCBs classified as cytochrome P450 (CYP450) enzyme inducers (Wolff et al. 1997). Therefore, we hypothesized that interactions may exist for these specific phthalate metabolites and PCB measures and their relationships with sperm motility. However, to fully explore the available data, we calculated the relative excess risk due to interaction (RERI) between all five phthalate monoesters [monomethyl phthalate (MMP), MEP, MEHP, MBP, and MBzP] and the six PCB measures [∑PCBs, PCB-138, PCB-153, and the three structure–activity groups of PCBs (Wolff et al. 1997)] in relation to all three semen parameters (i.e., sperm concentration, motility, and morphology). The three structure–activity groups of PCBs were, for group 1, potentially estrogenic and weak phenobarbital inducers (PCB congeners 44, 49, 52, 101, 187, 174, 177, and 157/201); group 2, potentially antiestrogenic and dioxin-like PCBs (PCB congeners 95/66, 74, 77/110, 105/141, 118, 156, 167, 128, 138, and 170); and group 3, phenobarbital and CYP1A and CYP2B inducers (PCB congeners 99, 153, 180, 196/203, and 183).

We performed multivariate logistic regression analyses to explore statistical interactions, in terms of both departures from additivity of relative risks (RRs) and significance of multiplicative interactions. Semen parameters were dichotomized based on the World Health Organization (WHO) reference values (WHO 1999) for sperm concentration (< 20 million/mL) and motility (< 50% motile sperm) and the strict criteria for morphology (< 4% normal sperm) (Kruger et al. 1988). Because our primary objective was to explore RR interactions, we dichotomized PCBs and phthalates at their median levels.

We used an approach described by Rothman (1986) to explore departure from RR additivity, as follows: Let *A* and *A*′ denote values above and below the median, respectively, for PCBs, and let *B* and *B*′ denote values above and below the median, respectively, for phthalates. Let RR(*AB*′), RR(*A*′*B*), and RR(*AB*) denote the RR of having semen parameters below reference value for the groups of subjects with high PCBs/low phthalates, low PCBs/high phthalates, and high PCBs/high phthalates, respectively, compared with the group of subjects with low PCBs/low phthalates (*A*′*B*′). RERI can be calculated as a measure of departure from additivity using the following equation (Rothman 1986):


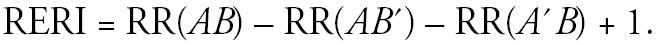


If there is no superadditive interaction (null hypothesis), RERI equals 0. If there is superadditivity, RERI is > 0, and subadditivity will yield an RERI < 0. Indicator variables for the exposure categories *AB*, *A′B*, and *AB* were created using the category *A*′*B*′ as the reference category. Odds ratios (ORs) were estimated by exponentiating the corresponding coefficients from the multiple logistic regression model. The ORs were substituted for the RRs in the RERI equation. Confidence intervals (CIs) for the RERI were calculated using bootstrap percentile 1 methodology recommended by Assmann et al. (1996). For example, for a 95% CI these are essentially the lower 2.5th and upper 2.5th percentiles of the empirical distribution of RERIs calculated from 500 data sets resampled with replacement from the original data.

To explore the significance of a multiplicative interaction between the dichotomized phthalate and PCB levels, a standard interaction term was included in the logistic regression models. Statistical significance of the interaction term was determined by calculating *p*-values based on Wald tests for the multiplicative interaction term (Hosmer and Lemeshow 1989). In addition, the predicted probabilities of below reference semen parameters were calculated from the logistic regression models that included both main effects of the phthalates and PCBs and their multiplicative interaction.

Covariates considered for inclusion in the multivariate logistic models included smoking status, age, and abstinence time (Blackwell and Zaneveld 1992; Kidd et al. 2001; Vine et al. 1994). Their final inclusion in the multivariate models was based on statistical and biologic considerations (Hosmer and Lemeshow 1989). Age was modeled as a continuous independent variable. Abstinence time was modeled as an ordinal five-category variable (i.e., < 2, 3, 4, 5, and ≥6 days). Smoking status was included as a dummy variable (current and former vs. never). All statistical analyses were conducted using SAS software, version 8.2 (SAS Institute Inc., Cary, NC).

## Results

### Participants and semen parameters.

Demographic characteristics by sperm motility are described in Table 1 for the 303 study subjects. An additional 25 subjects with semen and PCB measures were excluded from the statistical analysis because they lacked phthalate measurements. Subjects were primarily white (82%) and had a mean (± SD) age of 36.0 ± 5.4 years, and 72% had never smoked. Using the WHO reference values (WHO 1999), 53 men (17%) had low sperm concentrations, 145 men (48%) had low percentages of motile sperm, and 78 men (26%) had abnormal sperm morphology. The semen parameter categories were not mutually exclusive (i.e., a man could be classified as having below reference values for more than one of the sperm parameters). Subjects having below reference sperm motility tended to be older than those having above reference sperm motility (Wilcoxon rank sum test, *p* = 0.02). There was no clear association of sperm concentration, motility, or morphology with either abstinence time or smoking status.

### Phthalate monoesters and PCBs.

There was a wide distribution of both PCBs and phthalate monoester levels (Tables 2 and 3, respectively). The rank order of the phthalate distribution was similar to the distribution found in NHANES 1999–2000 (Silva et al. 2004), with the highest median levels found for MEP (148 ng/mL), followed by MBP (15.1 ng/mL), MBzP (7.0 ng/mL), MEHP (5.4 ng/mL), and MMP (4.5 ng/mL). MMP was not measured in the NHANES 1999–2000 population. The median concentration of the ∑PCBs was 212 ng/g lipid.

### Interaction analyses.

To explore the main effects of the phthalate monoesters and the PCBs with semen parameters, separate logistic regression models were fitted in which each semen parameter was regressed on pairs of one phthalate and one PCB in each model. Although models were run for each semen parameter, only models for sperm motility are presented because they had the strongest associations with phthalate monoesters and PCBs. There was an association between below reference value sperm motility and MBP above the median (OR = 1.65; 95% CI, 1.04–2.62) and a suggestive association between sperm motility and PCBs classified as CYP450 enzyme inducers (OR = 1.38; 95% CI, 0.84–2.27). These ORs represent the ORs for MBP (or enzyme-inducing PCBs) pooled over both high and low levels of the other analyte. There were suggestive relationships between sperm motility and the ∑PCBs (OR = 1.48; 95% CI, 0.90–2.43) and PCB-153 (OR = 1.41; 95% CI, 0.86–2.29). In models with MBzP and PCBs, there was no association of sperm motility with MBzP (OR = 1.07; 95% CI, 0.67–1.69) but suggestive associations with the ∑PCBs (OR = 1.51; 95% CI, 0.92–2.46), PCB-153 (OR = 1.44; 95% CI, 0.89–2.34), and PCBs classified as CYP450 enzyme inducers (OR = 1.41; 95% CI, 0.86–2.32). Although these pooled ORs may be non-significant in the main effect models, it should be noted that such estimates may not be appropriate summary measures of effects in the presence of interactions.

In the models exploring the additive interactions between dichotomized phthalates and PCBs, both crude and adjusted ORs for low sperm motility were calculated (Table 4). The crude ORs were nearly identical to the adjusted ORs (coefficients were within 5%) and therefore are not presented. After adjustment for age and abstinence time, men with MBP levels above the median and the sum of enzyme-inducing PCB levels below the median (high MBP/low PCB) and men with MBP levels below the median and the sum of enzyme-inducing PCB levels above the median (low MBP/high PCB) were not more likely to have sperm motility values below the WHO reference value (low MBP/low PCB) (OR = 1.0; 95% CI, 0.55–1.86 and OR = 0.89; 95% CI, 0.47–1.67, respectively) than men with both MBP and the sum of enzyme-inducing PCB levels below the median. In contrast, men with both MBP and the sum of enzyme-inducing PCB levels above the median (high MBP/high PCB) were 2.77 times (95% CI, 1.47–5.19) more likely to have sperm motility values below the reference value than men with low MBP/low PCBs.

After adjustment for age and abstinence time, there was a greater than additive interaction between MBP and sum of enzyme-inducing PCBs (RERI = 1.87; 95% CI, 0.56–4.52) for the risk of below reference sperm motility. There was also an interaction between MBP and PCB-153 (RERI = 1.42; 95% CI, 0.09–3.76) and a suggestive interaction between MBP and ∑PCBs (RERI = 1.35; 95% CI, −0.11 to 3.48). Similarly, for MBzP, there were interactions with sum of CYP450 enzyme-inducing PCBs (RERI = 1.30; 95% CI, 0.21–3.06), PCB-153 (RERI = 1.40; 95% CI, 0.41–3.22), and the ∑PCBs (RERI = 1.24; 95% CI, 0.15–2.94) (Table 4). There were no significant interactions between the other phthalates (MEP, MMP, and MEHP) and PCBs (PCB-118, PCB-138, PCB-153, and the three groupings of PCBs) with sperm motility. Similarly, there were no significant interactions between any phthalates and PCBs with sperm concentration or morphology.

Typically, the magnitudes of interactions that are detected as consistent with a multiplicative interaction term are much larger than those that might be statistically detected as greater than additive. However, in our analysis we found that evaluation of interaction on the multiplicative scale yielded results very similar to those of the RERI measures and their significance. After adjusting for age and abstinence time, for below reference sperm motility values, there were multiplicative interactions between MBP and PCB-153 (*p*-value = 0.07) and group 3 PCBs (*p*-value = 0.008), as well as interactions between MBzP and the ∑PCBs (*p*-value = 0.02), PCB-153 (*p*-value = 0.004), and group 3 PCBs (*p*-value = 0.01). There was a suggestive multiplicative interaction between MBP and ∑PCBs (*p*-value = 0.12).

The predicted percentages of men with below reference sperm motility based on the interactions between levels of MBP and MBzP with PCBs are presented in Table 5. The table shows the percentage of subjects with below reference sperm motility; it does not show the percentage of motile sperm. For instance, the predicted percentages of men with below reference sperm motility values by MBP and group 3 PCBs were 45% for subjects with low MBP/low group 3 PCBs, 37% for subjects with low MBP/high group 3 PCBs, 42% for subjects with high MBP/low group 3 PCBs, and 65% for subjects with high MBP/high group 3 PCBs.

## Discussion

In the present study, we found evidence of interactions between some PCBs and phthalates in relation to alterations in human sperm motility. Specifically, we found interactions between MBP and MBzP with enzyme-inducing PCBs, ∑PCBs, and PCB-153 in relation to sperm motility. We recognize that this was an exploratory analysis that involved multiple comparisons. However, because this was one of the first studies to explore interactions among these families of chemicals, we felt it was important to examine all potential phthalate monoesters and PCB category combinations even though our *a priori* hypothesis for interactions included only MBP, MBzP, ∑PCBs, and PCBs classified as CYP450 enzyme inducers.

One potential limitation of our analysis method is that we grouped the levels of both phthalates and PCBs into “low” and “high” levels based on their medians in order to take advantage of the RERI approach for evaluating greater than additive interaction. As a result, it is hypothetically possible that the interactions observed could be an artifact of higher underlying levels of one compound at the “high” level of the other compound compared with the “low” level of the other compound. However, for our study we found that this was not the case; the mean underlying levels of MBP and MBzP were essentially equivalent between low and high levels of each of the three PCB groups, and the mean underlying levels of the groups of PCBs, although slightly higher for “high” MBP (or MBzP) than for “low” MBP (or MBzP) within the “high” PCB groups, were never significantly higher. In addition, the interaction between MBzP and each of the PCB measures (∑PCBs, PCB-153, and enzyme-related PCBs) was statistically significant even when the underlying continuous measures were used as main effects and multiplicative interactions, supporting the validity of our analysis.

Although we are not aware of animal or human data on the interaction of phthalates and PCBs in relation to sperm motility, there are data on their independent relationships with sperm motility. Bush et al. (1986) found significant inverse relationships between sperm motility and seminal plasma concentrations of PCB congeners 153, 138, and 118. Dallinga et al. (2002) analyzed serum for PCB congeners 118, 138, 153, and 180 and their metabolites and found an inverse relationship between the sum of PCB metabolites and sperm progressive motility and concentration. In another recently published study, Richthoff et al. (2003) found weak but statistically significant negative associations between PCB-153 and sperm motility. In our previous publication (Hauser et al. 2003), we found evidence of inverse associations between PCB-138, ∑PCBs, and PCBs classified as enzyme inducers, and sperm motility and morphology.

OH-PCBs, formed by CYP1A and CYP2B during the phase I metabolism of PCBs, have been found in human blood at levels comparable with the parent PCBs (Bergman et al. 1994; Fangstrom et al. 2002; Hovander et al. 2002; Letcher et al. 2000). Although we did not measure OH-PCBs in the present study, future plans include measuring OH-PCBs, potentially strengthening the relationships previously found between some groups of PCBs and semen parameters (Hauser et al. 2003), as well as potentially strengthening the interactions found between some groups of PCBs and phthalates in the present study.

Toxicologic studies, primarily in rats, consistently show that select phthalate monoesters, including MEHP, MBzP, and MBP, are male reproductive toxicants (Cater et al. 1977; Foster et al. 1981; Oishi 1986; Park et al. 2002). Testicular toxicity, manifesting as testicular atrophy and spermatogenic cell loss, was found after phthalate exposure. The Sertoli cell is considered the primary target of phthalate monoester toxicity. Phthalate monoesters affect the normal nurse function of the Sertoli cell and its ability to produce lactate and pyruvate from glucose, an essential function of the Sertoli cell in the sustenance of germ cells, which cannot use glucose as an energy substrate (Williams and Foster 1988). Studies have also shown that select phthalate monoesters may interfere with the ability of Sertoli cells to respond to their normal endogenous ligand, follicle-stimulating hormone (FSH). MBP and MEHP inhibit the ability of the FSH to stimulate its normal cellular second messenger, cAMP (Heindel and Chapin 1989; Heindel and Powell 1992; Lloyd and Foster 1988). These studies showed that the site of action of the phthalate monoester was at the coupling of the FSH receptor–ligand complex to the transducing G-protein within the Sertoli cell membrane. Dimethyl and diethyl phthalate were without Sertoli cell effects even at high dose levels (Heindel and Powell 1992). Phthalate monoester toxicity has also been found to be associated with an increased rate of apoptosis of germ cells (Richburg and Boekelheide 1996), which may be partially responsible for the loss of spermatogenic cells in the testis.

Because of the ongoing recruitment of participants for our research, the subjects in the present study overlap with the subjects in our previous studies on the independent associations between exposure to PCBs (Hauser et al. 2003) and phthalates (Duty et al. 2003) with human semen parameters. Because all subjects in our studies were partners of an infertile couple, the generalizability of our results to men in the general population remains unclear because men visiting an infertility clinic may be more susceptible to environmental chemicals than men from the general population. However, there is currently no evidence to support or refute the hypothesis that men attending an infertility clinic are more susceptible to PCBs or phthalates than any other group of men.

## Conclusion

An understanding of how chemical classes interact is essential to determining risk because humans are concurrently exposed to numerous classes of chemicals, including phthalates and PCBs (CDC 2003). In the present study, we found statistical interactions between some PCBs and phthalates in relation to low sperm motility. Although our study was not designed to determine the biologic mechanism(s) underlying statistical interactions, we hypothesize that the mechanism is through interactions between PCB metabolites and enzymes responsible for phthalate metabolism, specifically UDP-GT. Our future work will include measures of PCB metabolites, as well as levels of free and glucuronidated phthalate monoesters in urine. This will allow us to directly explore the potential inhibitory action of PCB metabolites on the glucuronidation of phthalate monoesters.

If there are interactions between these two ubiquitous classes of chemicals, risk assessments for human exposure to phthalates would need to be revised to account for a potentially increased risk of altered semen quality in the presence of exposure to PCBs. The risk assessment and public health implications of an interaction between these two ubiquitous classes of compounds make it important to conduct further human studies to confirm these results and identify potential mechanisms of interactions.

## Correction

Because of differences due to rounding in earlier analyses, values for RERI, OR, and 95% CI were incorrect in the “Abstract” and the text of the original manuscript published online but were correct in Table 4. These values have been corrected here.

## Figures and Tables

**Table 1:** Demographic characteristics by sperm motility (*n* = 303).

	Sperm motility		
	Total subjects (*n* = 303)	≥50% motile (*n* = 158)	< 50% motile (*n* = 145)
Age			
Median (years)	35.0	35.0	36.0
Categories [*n* (%)]			
≤30 years	39 (13)	25 (16)	14 (10)
31–35 years	114 (38)	70 (44)	44 (30)
36–40 years	96 (32)	38 (24)	58 (40)
> 41 years	54 (18)	25 (16)	29 (20)
Abstinence time [*n* (%)]			
≤2 days	73 (24)	39 (25)	34 (23)
3 days	96 (32)	49 (31)	47 (32)
4 days	51 (17)	25 (16)	26 (18)
5 days	27 (9)	16 (10)	11 (8)
≥6 days	54 (18)	28 (18)	26 (18)
Unknown	2 (1)	1 (1)	1 (1)
Smoking status [*n* (%)]			
Never smoker	217 (72)	115 (73)	102 (70)
Ever smoker	83 (27)	42 (27)	41 (28)
Unknown	3 (1)	1 (1)	2 (1)

Pearson’s chi-square test for smoking status, *p*-value = 0.71; Mantel-Haenszel chi-square test for trend for abstinence time, *p*-value = 0.95 (subjects with unknown smoking status or abstinence time were excluded from the chi-square tests); Wilcoxon rank sum test for age, *p*-value = 0.02.

**Table 2:** Distribution of serum levels (in ng/g lipid) of PCB-118, PCB-138, PCB-153, and ∑PCBs, and structure–activity groupings of PCBs (*n* = 303).

	Percentile					
	5th	25th	50th	75th	95th	Geometric mean
PCB-118	4.4	7.6	11.9	18.5	35.2	12.1
PCB-138	14.0	22.2	31.2	47.8	102	33.4
PCB-153	17.5	27.7	40.9	60.4	125	43.0
∑PCB congeners	92.3	152	212	312	592	223
∑Estrogenic PCBs (group 1)	6.8	10.6	15.5	22.1	47.8	16.1
∑Dioxin-like PCBs (group 2)	36.9	54.8	74.3	117	227	80.6
∑Enzyme-inducing PCBs (group 3)	36.3	59.9	87.8	133	263	91.8

**Table 3:** Distribution of urinary phthalate monoester concentrations (in ng monoester/mL urine) unadjusted and adjusted for specific gravity (*n* = 303).

	Percentile					
	5th	25th	50th	75th	95th	Geometric mean
Unadjusted						
MEP	16.9	48.7	148	514	1,937	160
MBP	2.3	7.8	15.1	31.8	75.1	14.7
MBzP	< LOD	2.9	7.0	14.4	35.0	6.3
MEHP	< LOD	2.0	5.4	20.6	131	5.9
MMP	< LOD	1.7	4.5	10.9	32.1	3.9
Specific gravity adjusted						
MEP	25.0	60.0	165	528	1,974	185
MBP	3.6	10.3	16.3	31.0	68.2	17.0
MBzP	< LOD	4.1	8.0	14.3	37.6	7.3
MEHP	< LOD	2.4	6.6	20.4	112	6.9
MMP	< LOD	2.2	5.0	11.8	32.5	4.5

LOD, limit of detection (MBzP = 0.5 ng/mL; MEHP = 0.9 ng/mL; MMP = 0.7 ng/mL).

**Table 4:** Adjusted*^a^* ORs (95% CIs) for low sperm motility, and RERI for the additive interactions between two phthalates (MBP and MBzP) and three categories of PCBs (enzyme-inducing PCBs, ∑PCBs, and PCB-153).

	Exposure level			OR by PCB category		
Phthalate monoester	Phthalate*^b^*	PCB*^b^*	Nos.*^c^*	Enzyme-inducing PCBs	∑PCBs	PCB-153
MBP	Low	Low	80, 80, 80	1.0	1.0	1.0
	High	Low	71, 71, 71	1.00 (0.55–1.86)	1.26 (0.68–2.34)	1.21 (0.66–2.23)
	Low	High	72, 72, 72	0.89 (0.47–1.67)	1.19 (0.63–2.24)	1.07 (0.57–2.01)
	High	High	80, 80, 80	2.77 (1.47–5.19)	2.80 (1.50–5.23)	2.68 (1.44–4.99)
	RERI			1.87* (0.56–4.52)	1.35 (^−^0.11–3.48)	1.42* (0.09–3.76)
MBzP	Low	Low	69, 73, 70	1.0	1.0	1.0
	High	Low	82, 78, 81	0.73 (0.40–1.34)	0.73 (0.39–1.34)	0.68 (0.37–1.26)
	Low	High	83, 79, 82	1.00 (0.54–1.85)	1.07 (0.57–2.00)	0.94 (0.51–1.75)
	High	High	69, 73, 70	2.03 (1.05–3.92)	2.03 (1.08–3.85)	2.03 (1.06–3.89)
	RERI			1.30* (0.21–3.06)	1.24* (0.15–2.94)	1.40* (0.41–3.22)

a Adjusted for age and abstinence time.

b MBP, MBzP, and PCBs were dichotomized at their median: “low” indicates exposure below the group median, and “high” indicates exposure above the group median.

c Numbers of subjects in each of the paired phthalate and PCB categories, respectively.

* *p* < 0.05.

**Table 5:** Adjusted*^a^* predicted percentages of men with below-reference sperm motility by high or low levels of two phthalates (MBP and MBzP) and three categories of PCBs (enzyme-inducing PCBs, ∑PCBs, and PCB-153).

	Exposure level			Predicted percentages by PCB category		
Phthalate monoester	Phthalate*^b^*	PCB*^b^*	Nos.*^c^*	Enzyme-inducing PCBs	∑PCBs	PCB-153
MBP	Low	Low	80, 80, 80	45	41	42
	High	Low	71, 71, 71	37	42	40
	Low	High	72, 72, 72	42	44	44
	High	High	80, 80, 80	65	63	63
MBzP	Low	Low	69, 73, 70	50	49	50
	High	Low	82, 78, 81	43	44	42
	Low	High	83, 79, 82	37	36	36
	High	High	69, 73, 70	60	60	61

a Adjusted for age and abstinence time.

b MBP, MBzP, and PCBs were dichotomized at their median: “low” indicates exposure below the group median, and “high” indicates exposure above the group median.

c Numbers of subjects in each of the paired phthalate and PCB categories, respectively.
